# Serum Calcium and the Risk of Breast Cancer: Findings from the Swedish AMORIS Study and a Meta-Analysis of Prospective Studies

**DOI:** 10.3390/ijms17091487

**Published:** 2016-09-06

**Authors:** Wahyu Wulaningsih, Harkiran K. Sagoo, Mustafa Hamza, Jennifer Melvin, Lars Holmberg, Hans Garmo, Håkan Malmström, Mats Lambe, Niklas Hammar, Göran Walldius, Ingmar Jungner, Mieke Van Hemelrijck

**Affiliations:** 1 Division of Cancer Studies, Cancer Epidemiology Group, King’s College London, London SE1 9RT, UK; wahyu.wulaningsih@kcl.ac.uk (W.W.); harkiran.sagoo@kcl.ac.uk (H.K.S.); mustafa.hamza@kcl.ac.uk (M.H.); jennifer.melvin@kcl.ac.uk (J.M.); lars.holmberg@kcl.ac.uk (L.H.); hans.garmo@kcl.ac.uk (H.G.); 2 Department of Surgical Sciences, Uppsala University Hospital, Uppsala 751 85, Sweden; 3 Regional Cancer Centre, Uppsala 751 83, Sweden; mats.lambe@ki.se; 4 Unit of Epidemiology, Institute of Environmental Medicine, Karolinska Institutet, Stockholm 171 77, Sweden; hakan.malmstrom@ki.se (H.M.); niklas.hammar@ki.se (N.H.); 5 Department of Medical Epidemiology and Biostatistics, Karolinska Institutet, Stockholm 171 77, Sweden; 6 AstraZeneca R&D, Mölndal 431 50, Sweden; 7 Unit of Cardiovascular Epidemiology, Institute of Environmental Medicine, Karolinska Institutet, Stockholm 171 77, Sweden; goran.walldius@ki.se; 8 Department of Medicine, Clinical Epidemiological Unit, Karolinska Institutet and CALAB Research, Stockholm 171 77, Sweden; ingmar.jungner@ki.se

**Keywords:** calcium, breast cancer, albumin, prospective study

## Abstract

To investigate the association between serum calcium and risk of breast cancer using a large cohort and a systematic review with meta-analysis. From the Swedish Apolipoprotein Mortality Risk (AMORIS) Study we included 229,674 women who had baseline measurements of serum total calcium and albumin. Multivariable Cox regression was used to assess the association between total and albumin-corrected calcium and breast cancer risk. For the systematic review, an electronic search of MEDLINE and EMBASE databases was performed to identify other prospective cohorts assessing the relationship between serum calcium and breast cancer risk. We pooled the results of our AMORIS cohort with other eligible studies in a meta-analysis using a random effects model. *I*^2^ test was used to assess heterogeneity. In the AMORIS study, 10,863 women were diagnosed with breast cancer (mean follow-up: 19 years). We found an inverse association between total serum calcium and breast cancer when comparing the fourth quartile to the first quartile (HR: 0.94, 95% CI: 0.88–0.99, *p* value for trend 0.04) and similar results using albumin-corrected calcium. In the systematic review, we identified another two prospective cohorts evaluating pre-diagnostic serum total calcium and breast cancer. Combining these studies and our findings in AMORIS in a meta-analysis showed a protective effect of serum calcium against breast cancer, with a summary RR of 0.80 (95% CI: 0.66–0.97). No substantial heterogeneity was observed. Our findings in AMORIS and the meta-analysis support an inverse association between serum calcium and breast cancer risk, which warrants mechanistic investigations.

## 1. Introduction

There is ongoing debate surrounding the role of calcium in cancer prevention. In the context of breast cancer, current findings are mostly focused on dietary calcium with limited evidence on biomarker levels, denoting an unclear role of the calcium metabolism. For instance, an inverse association with dietary calcium intake was shown in a meta-analysis including fifteen observational studies [1], with an approximate 19% reduction in breast cancer risk observed among those in highest quantile of calcium intake compared to the lowest quantile. This protective effect is thought to be majorly underlain by chemopreventative actions of 1,25-dihydroxyvitamin D (1,25(OH)2D), the active form of vitamin D, a well-known regulator of calcium. In experimental studies, 1,25(OH)2D has been shown to induce differentiation and apoptosis and inhibit cellular proliferation and angiogenesis in normal and malignant breast cells [2,3,4]. Similar associations were reported in observational studies, where a meta-analysis showed that levels of circulating 1,25(OH)2D inversely correlated with risk of breast cancer [5]. On the other hand, evidence on the role of circulating calcium levels in breast cancer is limited [6,7].

In addition to 1,25(OH)2D, calcium metabolism is also regulated by other hormonal factors, such as parathyroid hormone (PTH), which altogether maintain serum ionized calcium, the active form, within a tight physiological range of approximately 10% [8]. Nevertheless, most observational studies focusing on the role of calcium in breast cancer utilized dietary calcium rather than its circulating levels, which may better reflect the extent of calcium homeostasis. In order to elucidate the role of circulating calcium levels in breast cancer development, we investigated the association between prediagnostic serum calcium and breast cancer risk in a large prospective cohort study including more than 220,000 women. Additionally, we systematically reviewed evidence from observational studies on the association between serum calcium and risk of breast cancer and carried out a meta-analysis for prospective studies to further clarify any association.

## 2. Results

### 2.1. The AMORIS Study

Characteristics of study participants are displayed in Table 1. A total of 10,863 (4.73%) women were diagnosed with breast cancer during follow-up (mean: 19 years). Mean calcium level was 2.38 mmol/L (SD: 0.10; range: 1.00 to 3.72) and mean albumin level was 42.50 g/L (SD: 2.74; range: 21 to 57). The majority (>95%) of the participants had serum calcium and albumin within normal range based on the laboratory cut-offs. When comparing the lowest and highest quartiles of serum calcium, women in highest quartiles of total and albumin-corrected calcium had greater age, higher education levels, and more co-morbidities. In univariable analysis of serum calcium, each covariate showed significant correlation with serum calcium (Table S1).

When assessing total serum calcium in relation to breast cancer risk, we found that higher calcium corresponded to a lower risk of breast cancer, with a hazard ratio (HR) for breast cancer of 0.78% and 95% confidence intervals (CI) of 0.63 to 0.97. This inverse trend was also seen when analyzing total calcium using its quartiles (HR: 0.94 (95% CI: 0.88–0.99) for the fourth compared to the first quartile, *p*-value for trend 0.04). Similarly, an inverse association was observed when albumin-corrected calcium was assessed (HR: 0.93 (95% CI: 0.88–0.99) for the fourth compared to the first quartile, *p*-value for trend 0.05). When serum albumin was assessed, no association with breast cancer risk was found (HR: 0.99, 95% CI: 0.98–1.01 for every g/L increase in albumin). On the other hand, evaluation of age- and sex-specific categories of calcium did not show any clear association (Table 2), which may be driven by a lack of precision. We additionally conducted a sensitivity analysis excluding those with follow-up time less than two years and similar findings were obtained, e.g., HR: 0.93 (95% CI: 0.88–0.99) for the fourth quartile of calcium when adjusted for albumin, Ptrend = 0.03. Results did not alter when we only included participants with calcium levels within normal range (results not shown). Associations between serum calcium and breast cancer were weaker in pre- and postmenopausal women separately when analyses were stratified using a cut-off age of 50 years (Table S2). Nevertheless, significantly lower risks of breast cancer with increasing calcium levels were observed among postmenopausal women (e.g., HR: 0.89, 95% CI: 0.82–0.97 for the fourth compared to the first quartile of calcium when adjusted for albumin).

### 2.2. Systematic Review

**Figure 1:**
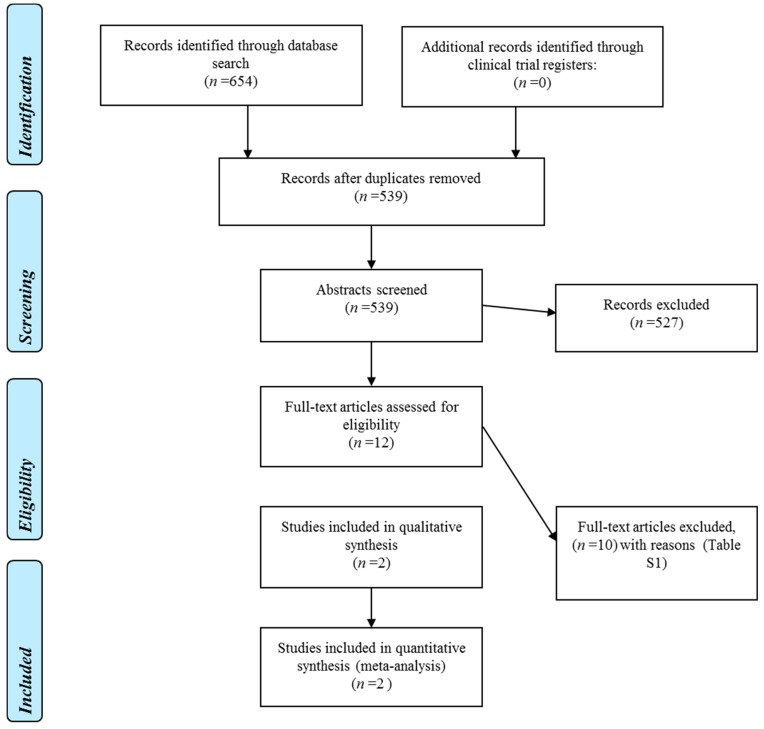
PRISMA diagram for study selection process for systematic review and meta-analysis.

Figure 1 displays the flow diagram of the search strategy used to select studies in this systematic review. We identified 654 potentially relevant articles. After excluding duplicates, titles, and abstracts were screened for the remaining 539 articles. Among these, we excluded 527 studies that did not satisfy the eligibility criteria based on information in the title and abstract, and articles published based on previously published studies. Full text was obtained and screened for 12 articles, and further 10 articles were excluded with reasons summarized in Table S3 (Supplementary Materials). Two articles that met the inclusion criteria were included in the qualitative assessment and meta-analysis along with the results of our AMORIS cohort.

**Figure 2:**
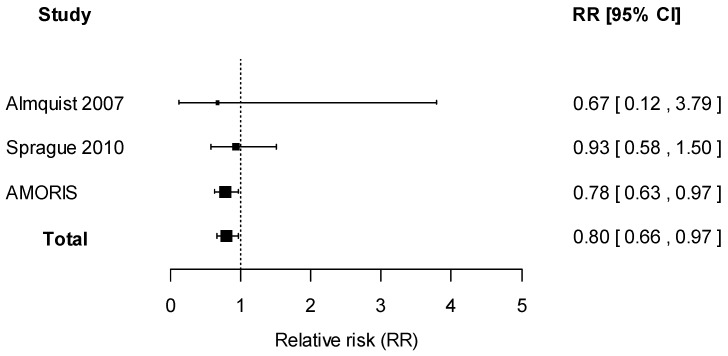
Forest plot for meta-analysis of the association between serum calcium and risk of breast cancer, expressed as relative risk of breast cancer for every 1 mmol/L increase in serum total calcium. Estimates from studies assessing serum calcium as categories were obtained from categorical regression.

Description of eligible studies identified from literature search is displayed in Table 3. Similar to the AMORIS study, all included studies were prospective cohorts with serum total calcium assessed prior to follow-up and any diagnosis of breast cancer. A study performed by Almquist and colleagues were based in Sweden, and the remaining study was conducted in the U.S. Apart from the AMORIS study, a lack of association between serum total calcium and breast cancer risk was reported by all studies, with estimates suggesting weak inverse associations [6,9]. All studies reported associations between serum calcium and breast cancer risk using serum calcium as categories. Although all contacted authors responded, they reported that estimates based on serum calcium as a continuous variable were no longer available. In one study by Sprague and colleagues, all women in the highest quartile of calcium had clinically high calcium levels [6], further rendering direct estimate summary between studies inappropriate. Therefore, using reported findings from categories, we performed categorical regression to estimate the linear relationship between serum calcium and breast cancer risk. Similar to findings using categories of calcium, inverse associations were suggested but no statistically significant results were observed (Figure 2).

#### 2.2.1. Methodological Quality

Quality assessment of the selected studies did not identify possible biases that could compromise the internal validity of the study leading to misinterpretation of the results. The longest follow-up duration was a mean of 19.40 years from the AMORIS cohort. Serum calcium was measured with standard laboratory procedures in all included studies. Each of studies included in our qualitative and quantitative analysis have described the adjustments made for potential confounding factors. Adjustments for age at baseline, educational level, and parity were performed in all included studies. Additionally, the studies identified in the systematic review aside from the AMORIS cohort also adjusted for additional breast cancer risk factors such as age at menarche and body mass index.

#### 2.2.2. Data Synthesis

A meta-analysis including the four studies reporting the association between serum calcium and breast cancer risk was performed using random-effects models. As shown in Figure 2, an inverse association was found, with an overall hazard ratio (HR) of 0.80% and 95% confidence intervals (CI) of 0.66 to 0.97. When assessing heterogeneity, no indication of heterogeneity was found (*I*^2^ = 0%). A funnel plot assessing the risk of publication bias showed symmetric distribution, indicating a lack of publication bias (Figure S1). A statistical test of asymmetry was not conducted because of the small number of included studies.

## 3. Discussion

In the AMORIS cohort, we found that higher serum total calcium contributed to a lower risk of breast cancer, particularly among women aged fifty and older. Combining our results in AMORIS and the additional three studies which satisfied our criteria in a meta-analysis, we found that higher serum calcium levels corresponded to a lower risk of breast cancer in the three prospective cohorts. There was no indication of publication bias as shown by the symmetrical funnel plot.

Evidence of a protective effect of calcium against breast cancer mostly rose from observational studies assessing dietary calcium intake [1]. A randomised control trial showed that calcium plus vitamin D supplementation did not reduce the overall risk of benign proliferative breast disease, a precursor to breast cancer, in postmenopausal women [10]. A similar lack of findings was apparent when looking at calcium plus vitamin D supplementation and the incidence of invasive breast cancer in postmenopausal women [11]. The beneficial effects of dietary calcium in reducing the risk of breast cancer as reported from population studies vary with menopausal status [2] and a moderately stronger association has been observed in premenopausal compared to postmenopausal women [12,13]. In a large prospective cohort study, higher dietary calcium intake corresponded to a lower risk of breast cancer in postmenopausal women, although confounding by use of calcium supplements in postmenopausal women taking hormone replacement therapy was evident [14]. Nevertheless, calcium levels are tightly regulated in the body rather than dependent on nutrient intake [8]. Confirming the suggested protective effect of calcium against breast cancer, we have now shown that circulating calcium, which is involved in many cellular processes [15], was inversely correlated to the risk of developing breast cancer. Such protective effect could be explained by increased cytosolic levels of calcium following higher serum calcium [8] which may affect multiple cellular processes including cell cycle and apoptosis, possibly via Ras and β-catenin pathways [16].

To our knowledge, this is the largest population-based study describing pre-diagnostic serum calcium levels and the risk of breast cancer in women, and the first systematic review describing this association. Results from our meta-analysis confirmed our findings in AMORIS, with higher pre-diagnostic serum calcium levels suggested to be associated with a lower risk of breast cancer in women. The calcium metabolism may be involved in carcinogenesis given its link to vitamin D, which has been linked to breast cancer cell proliferation [2,4]. However, calcium plays a direct role in cell apoptosis [17]. In cancer cells, abnormal expression of Ca^2+^ pumps and channels impairs calcium homeostasis promoting tumor growth and migration, overcoming apoptosis [17]. Therefore, ionized calcium levels may predict breast cancer risk, since total calcium levels also account for the calcium bound to protein. On the other hand, levels of calcium are also influenced by tumor growth among breast cancer patients, suggesting potential reverse causation [18]. However, our study and other studies included in the meta-analysis were based on serum calcium measured in samples collected prior to any diagnosis of breast cancer.

Existing literature discussing pre-diagnostic serum calcium levels and the risk of breast cancer is limited. In addition to a role in breast cancer incidence, pre-diagnostic calcium levels have additionally been reported to be associated with breast cancer mortality. Huss and colleagues [19] reported an inverse relation between pre-diagnostic levels of serum calcium and breast cancer mortality, with lower breast cancer-specific mortality in patients with serum calcium levels ≥2.44 mmol/L compared to those with lower levels of calcium (HR: 0.53, 95% CI: 0.30–0.92). However, this association was no longer seen in a sensitivity analysis excluding women diagnosed with breast cancer within two years from the date of baseline blood donation. The association between calcium and breast cancer death is beyond the scope of the current study. Nevertheless, considering the inverse association observed between calcium levels and breast cancer, it would be of interest to further explore any clinical role of serum calcium in breast cancer diagnosis and prognosis.

Our analyses in the AMORIS study were strengthened by the large number of women with measurements of calcium and albumin conducted in the same clinical facility. The study population in AMORIS included samples from non-hospitalized women. However, the use of a healthy cohort does not affect the internal validity of our study. Our study is limited by the lack of information on free ionized calcium. Nevertheless, albumin-corrected serum calcium is considered a valid assessment of ionized calcium on a group level [20]. There was also a lack of information regarding lifestyle-related factors such as BMI and hormonal or reproductive factors apart from parity. To address this, we used age of fifty years as a cut-off for menopause and co-morbidities as a proxy of lifestyle-related disorders. Finally, we only used a single measurement of serum calcium rather than repeated measurements. This may have resulted in attenuation of observed associations.

The meta-analysis performed was strengthened by the quality of individual studies, which used standard assessments of serum calcium and breast cancer incidence. Additionally, we performed categorical regression allowing comparison of effects between studies assessing calcium as categories. A limitation of our meta-analysis was the low quantity of primary evidence available because only four studies, including our AMORIS study, were included. The primary data used in the meta-analysis was limited to populations from Sweden and the U.S. Nevertheless, similar to the AMORIS study, this does not affect the internal validity of individual studies. Although adjustment for potential confounders varied between included studies and may have affected effect size observed, similar directions of associations were seen in all studies. To further delineate the association between calcium and breast cancer, future studies should consider both total calcium levels and ionized calcium levels, which are metabolically active, as well as taking into account metabolic and endocrine regulators of the calcium metabolism, which may affect the risk of breast cancer.

## 4. Methods

### 4.1. The AMORIS Study

#### 4.1.1. Study Population

The Apolipoprotein Mortality Risk (AMORIS) Study has previously been described [21]. The cohort for this study consists of Swedish men and women recruited from the greater Stockholm area. Sequential blood sampling was performed for these participants at the Central Automation Laboratory (CALAB) in Stockholm, Sweden, during 1985–1996, for either routine check-ups or outpatient referrals. None of the participants were inpatients during the time of sampling [22]. From this cohort we identified 229,674 women aged ≥20 with baseline serum calcium and albumin and no previous history of breast cancer for inclusion in our study. The study complied with the Declaration of Helsinki and was approved by the ethics review board of the Karolinska Institute.

Details of breast cancer diagnosis were acquired from the Swedish National Cancer Register and the Stockholm Clinical Quality Register of Breast Cancer, which has 97% coverage and is validated in terms of the records of the National Cancer Register. The International Classification of Diseases version 7 (ICD-7) was used to identify breast cancer cases (ICD-7: 170). Follow-up time was defined as the period between baseline measurement and diagnosis of breast cancer, death, emigration, or end of study (31 December 2011), whichever occurred first.

Total serum calcium was measured using colorimetry (coefficient of variation <2.5%), and albumin was measured using a bromcresolgreen (BCG) method (coefficient of variation <1.8%) [20]. Levels of uncorrected calcium were categorized as low, normal, or high in terms of whether they were lower, equal to or higher than the reference intervals respectively, based on age and sex used by the CALAB laboratory (Panel 1). Calcium levels lower than 1 mmol/L and higher than 4 mmol/L were consider outliers based on the laboratory practice and were removed from the study population. Similar procedures were applied for albumin using a lower cut-off of 20 g/dL and upper cut-off point of 60 g/dL. In addition to total calcium, albumin-corrected calcium was calculated assuming normal albumin levels; for each 1 g/L that the albumin concentration was below 40 g/L (normal concentration), the calcium concentration was increased with 0.02 mmol/L [20]. Similar approach was performed for albumin concentration above 40 g/L. All methods were fully automated, calibrated and performed at the same accredited laboratory [21].

We also collected information on baseline age (years), parity (nulliparious, 1+) to account for reproductive risk factors, and categorized the level of education into low (primary school or less), moderate (high school) and high (higher education). There was a lack of information on potential lifestyle-related determinants of serum calcium including body mass index (BMI), smoking, and alcohol consumption [23] for all participants. Since these lifestyle factors are closely linked to other diseases such as cardiovascular disease, co-morbidities were assessed as a surrogate by calculating Charlson co-morbidity index (CCI) from the history of hospitalization. The CCI comprises 17 groups of diseases, with each disease category (1, 2, 3, and 6) carrying a specific weight. These weights were summated to generate an overall score of four co-morbidity levels, (0, 1, 2, and 3+) representing a scale ranging from no co-morbidity to severe co-morbidity. We additionally collected information on history of fractures from the inpatient register. Season at index examination (spring, summer, autumn, winter) was included as a proxy for vitamin D, since information on its serum levels was unavailable.

#### 4.1.2. Statistical Analysis

Multivariable Cox regression was used to assess the association between continuous levels and quartiles of total and albumin-corrected calcium in addition to age- and sex-specific categories of calcium. A test for trend was conducted by using assignment to categories as an ordinal scale. Adjustments were made across all models for age, education, parity, history of fractures, CCI, and season at index examination. The Cox proportional hazards models were also adjusted for albumin levels as a continuous variable when testing the association between uncorrected total calcium and breast cancer risk. Sensitivity analyses was conducted by excluding those with follow-up time less than two years (*n* = 3427) and only including those with calcium levels within normal range, i.e., 2.2–2.6 mmol/L (*n* = 222,128). Findings were stratified by age of 50 years as a proxy of menopause. Statistical significance was defined at two-sided *p*-value <0.05. All analyses were conducted with Statistical Analysis Systems (SAS) release 9.4 (SAS Institute, Cary, NC, USA).

### 4.2. Systematic Review

#### 4.2.1. Search Strategies

This review was conducted using guidance from the Cochrane Handbook for Systematic Reviews of interventions [24] and reported in line with the PRISMA guidelines [25]. An electronic search was performed to identify all original articles describing serum calcium and the risk of breast cancer. The search was limited to articles on human subjects and published in English only. MEDLINE and EMBASE databases were searched from their inception to 20 November 2015. The final search was performed on 20/11/2015. The following full search strategy was used: [cancer OR neoplasm *] AND breast AND [serum OR blood] AND calcium AND to “causation-etiology (best balance of sensitivity and specificity)”. Articles describing levels of serum calcium and the risk of breast cancer in women retrieved through this search were screened by two investigators and any disagreement was resolved by consensus. Reference lists of the included articles were hand-searched for potentially relevant articles.

Studies included were prospective population-based cohort which measured serum calcium levels at baseline and then reported breast cancer incidence during follow-up. Each included cohort study had to report either risk estimates (relative risks, odds ratios, or hazard ratios) with 95% confidence intervals (CI), or provide sufficient data to estimate these. We excluded publications when results from the same study had been published previously. The eligibility of each study was assessed by two independent investigators.

#### 4.2.2. Data Extraction

The following information was obtained from eligible studies: study design, population, risk estimates, and their 95% CIs. We extracted the relative risk for serum calcium as a continuous variable when available, and contacted study authors to request this data if not already described. All measurements were transformed into a common measurement unit (mmol/L) to allow comparability. For studies which only reported relative risk in categories e.g., quartiles, the covariance-corrected method of Greenland and Longnecker [26] was employed using the *dosresmeta* package in R version 3.1.2 (R Foundation for Statistical Computing, Vienna, Austria) to obtain linear dose-response estimates. The assignment of interval scores of serum calcium categories from the original studies were based on medians or means when available. Category midranges were applied for the remaining closed-ended categories. For upper open-ended categories with *b_i_* as the lower bound of the *i*th interval and the intervals indexed by *i* = 1, …, *n*, interval scores were assigned as *b_n_* + 0.5(*b_n_* − *b_n-_*_1_) [27]. Correspondingly, interval scores for the lower open-ended categories were assigned as *b*_2_ − 0.5(*b*_2_ − *b*_1_).

#### 4.2.3. Methodological Quality

We assessed the methodological quality of included studies using three components which may influence the strength of association between serum calcium and breast cancer risk: duration of follow-up, validity of serum calcium measurement, and extent of adjustments made for potential confounders.

#### 4.2.4. Data Synthesis

Qualitatively, we summarized and tabulated information about study participant characteristics, serum calcium measurements, length of follow-up, and definition of breast cancer diagnosis as the outcome. A random effects model using an inverse-variance method was used to obtain the average effect estimates of breast cancer risk by each unit increment in calcium levels. We evaluated heterogeneity with the *I^2^* statistic as a measure of the proportion of total variation in estimates attributable to random error, where any values >50% indicated substantial heterogeneity. Publication bias was examined with a funnel plot. Sensitivity analysis was performed for prospective cohort studies. All quantitative data synthesis was performed in RevMan version 5.3 (Copenhagen, Denmark).

## 5. Conclusions

In the AMORIS study, higher baseline serum calcium was associated with a lower risk of a breast cancer diagnosis. The meta-analysis confirmed this inverse association between calcium and subsequent breast cancer risk in prospective cohort studies. To elucidate pathways linking calcium and breast carcinogenesis the findings need to be confirmed in mechanistic studies and observational studies integrating information from dietary supplementation of calcium.

## Figures and Tables

**Table 1:** Characteristics of study population in AMORIS, overall, and for the lowest and highest serum calcium quartiles.

Serum Calcium (mmol/L)			
	All (*n* = 229,674)	Q1 < 2.31 (*n* = 55,357)	Q4 ≥ 2.44 (*n* = 59,462)
**Age (years)** Mean (SD)	46.16 (15.19)	44.49 (13.72)	48.82 (16.25)
**SES**			
High	82,555 (35.94)	21,707 (39.21)	18,882 (31.75)
Low	116,202 (50.59)	27,295 (49.31)	31,084 (52.28)
Unclassified	30,917 (13.46)	6355 (11.48)	9496 (15.97)
**Education Status**			
High	56,856 (24.76)	15,649 (28.27)	12,233 (20.57)
Middle	98,268 (42.79)	23,918 (43.21)	24,631 (41.42)
Low	62,456 (27.19)	13,182 (21.81)	18,775 (31.57)
Missing	12,094 (5.27))	2608 (4.71)	3823 (6.43)
**Parity**			
Yes	155,706 (67.79)	38,594 (69.72)	40,094 (67.43)
No	73,968 (32.21)	16,763 (30.28)	19,368 (32.57)
	**Breast Cancer**		
Yes	10,863 (4.73)	2656 (4.80)	2829 (4.76)
No	218,811 (95.27)	52,701 (95.20)	56,633 (95.24)
**Calcium (mmol/L)** Mean (SD)	2.38 (0.10)	2.26 (0.04)	2.50 (0.06)
**Corrected Calcium (mmol/L)** Mean (SD)	2.32 (0.09)	2.23 (0.06)	2.43 (0.08)
**Albumin (g/L)** Mean (SD)	42.50 (2.74)	41.15 (2.65)	43.76 (2.64)
**Charlson Comorbidity Index**			
0	213,122 (92.79)	51,713 (93.42)	54,205 (91.16)
1	8819 (3.84)	2023 (3.65)	2712 (4.56)
2	5692 (2.48)	1155 (2.09)	1870 (3.14)
3+	2041 (0.89)	466 (0.84)	675 (1.14)
**History of Fractures**			
Yes	173 (0.08)	45 (0.08)	43 (0.07)
No	229,501 (99.92)	55,313 (99.92)	59,419 (99.93)
**Seasonality**			
Spring	66,166 (28.81)	16,150 (29.17)	17,455 (29.35)
Summer	37,733 (16.43)	8996 (16.25)	10,150 (17.07)
Autumn	68,503 (29.83)	16,620 (30.02	16,924 (28.46)
Winter	57,272 (24.94)	13,591 (24.55)	14,933 (25.11)
**Follow-up (years)** Mean (SD)	19.40 (5.91)	19.58 (5.75)	18.99 (6.31)

**Table 2:** Association between serum total calcium (age-specific and albumin-corrected) and risk of breast cancer. All models are adjusted for age, education, parity, history of fractures, Charlson Comorbidity Index, and season at index examination.

	N (%)		Hazard Ratio (95% CI)
	Breast Cancer	No Breast Cancer	
**Calcium, mmol/L**			0.78 (0.63–0.97)
**Quartiles of calcium**			
<2.31	2656 (24.45)	52,701 (24.09)	1.00 (Reference)
2.31–2.36	2416 (22.24)	49,109 (22.44)	0.95 (0.90–1.00)
2.36–2.44	2962 (27.27)	60,368 (27.59)	0.96 (0.91–1.01)
≥2.44	2829 (26.04)	56,633 (25.88)	0.94 (0.88–0.99)
*p*-value for trend			0.04
**Calcium according to age-specific cut-offs**			
Low	128 (1.18)	2337 (1.07)	1.12 (0.94–1.34)
Normal	10,557 (97.18)	212,101 (96.93)	1.00 (Reference)
High	178 (1.64)	4373 (2.00)	0.94 (0.81-1.09)
*p*-value for trend			0.78
**Albumin-corrected calcium, mmol/L**			0.79 (0.63–0.99)
Quartiles of albumin-corrected calcium *			
<2.26	2320 (21.36)	49,316 (22.54)	1.00 (Reference)
2.26–2.32	2729 (25.12)	56,756 (25.94)	0.94 (0.89–1.00)
2.32–2.38	2697 (24.83)	53,093 (24.26)	0.96 (0.91–1.01)
≥2.38	3117 (28.69)	59,645 (27.26)	0.93 (0.88–0.99)
*p*-value for trend			0.05

* Model not adjusted for albumin.

**Table 3:** Description of studies included in the meta-analysis apart from the AMORIS study.

Study	Country	Study Design	Sample Size	Participant Characteristics	Follow-up Duration	Assessment of Serum Calcium	Findings			Adjustment
							Estimates	Total Calcium (mmol/L)	Breast Cancer Risk	
Almquist et al. 2007 [9]	Sweden	Cohort	7847	Mean age 52.3 years	Mean: 17.8 years	Serum total calcium by photometry	Relative risk (RR)	<2.29	Reference	Age, educational level, BMI, age at menarche, use of oral contraception, number of children, use of hormone-replacement therapy (HRT), smoking status, marital status, and alcohol consumption
								2.29–2.34	0.99 (0.76–1.28)	
								2.35–2.40	1.05 (0.81–1.36)	
								≥2.40	0.89 (0.67–1.19)	
Sprague et al. 2010 [6]	USA	Cohort	2338	Mean age 62.0 years	Mean: 14.3 years	Serum total calcium by ion-selective electrode analyzer	Hazard ratio (HR)	1.40–2.28	Reference	Age, education, menopausal status, age at menarche, age at menopause, parity, age at first birth, alcohol consumption, body mass index, and postmenopausal hormone use
								2.39–2.44	0.70 (0.42–1.17)	
								2.45–2.52	0.84 (0.51–1.40)	
								2.53–3.16	0.98 (0.60–1.60)	

## Supplementary Materials

Supplementary materials can be found at www.mdpi.com/1422-0067/17/9/1487/s1.
