# Supplementary material for: Serum Calcium and the Risk of Breast Cancer: Findings from the Swedish AMORIS Study and a Meta-Analysis of Prospective Studies
Source: Int J Mol Sci. 2016 Sep 6;17(9):1487. doi: 10.3390/ijms17091487 (PMC5037765; doi:10.3390/ijms17091487)
Supplement: Supplementary file 1 [file ijms-17-01487-s001.pdf]

Supplementary Materials: Serum Calcium and the Risk of Breast Cancer: Findings from the Swedish AMORIS Study and a Meta-Analysis of Prospective Studies

Wahyu Wulaningsih, Harkiran K. Sagoo, Mustafa Hamza, Jennifer Melvin, Lars Holmberg, Hans Garmo, Håkan Malmström, Mats Lambe, Niklas Hammar, Göran Walldius, Ingmar Jungner and Mieke Van Hemelrijck

Table S1. Univariable association between serum calcium (mmol/L) and participant characteristics.

	β (SE)	*p*-Value
**Age (years)—Mean (SD)**	2.34 (0.001)	<0.0001
**SES**		
High	Reference	<0.0001
Low	0.01 (0.0004)	
Unclassified	0.02 (0.0007)	
**Education Status**		
High	Reference	<0.0001
Middle	0.01 (0.0005)	
Low	0.02 (0.0006)	
Missing	0.02 (0.01)	
**Parity**		
Yes	Reference	<0.0001
No	0.003 (0.0004)	
**Albumin (g/L)—Mean (SD)**	1.82 (0.003)	<0.0001
**Charlson Comorbidity Index**		
0	Reference	<0.0001
1	0.01 (0.001)	
2	0.02 (0.001)	
3+	0.01 (0.002)	
**History of Fractures**		
Yes	Reference	<0.0001
No	2.38 (0.0002)	
**Seasonality**		
Spring	Reference	<0.0001
Summer	−0.0003 (0.0005)	
Autumn	0.001 (0.0006)	
Winter	−0.003 (0.0006)	

Table S2. Associations between calcium and breast cancer in AMORIS by menopausal status using age of 50 years. All models are adjusted for age, education, parity, history of fractures, CCI, and season at index examination.

	N (%)		Hazard Ratio (95%CI)
	Breast Cancer	No Breast Cancer	
**Age < 50**			
**Calcium, mmol/L**			1.20 (0.88–1.63)
**Quartiles of calcium**			
<2.31	1622 (28.27)	38062 (27.55)	1.00 (Reference)
2.31–2.36	1425 (24.84)	33122 (23.97)	1.04 (0.97–1.12)
2.36–2.44	1489 (25.95)	37619 (27.22)	1.02 (0.95–1.10)
≥2.44	1201 (20.93)	29376 (21.26)	1.05 (0.96–1.13)
p-value for trend			0.37
**Calcium according to age-specific cut-offs**			
Low	62 (1.08)	1333 (0.96)	1.01 (0.79–1.30)
Normal	5584 (97.33)	134336 (97.22)	1.00 (Reference)
High	91 (1.59)	2510 (1.82)	1.17 (0.95–1.44)
**Quartiles of albumin-corrected calcium ***			
<2.26	1585 (27.63)	38835 (28.10)	1.00 (Reference)
2.26–2.32	1610 (28.06)	39788 (28.79)	0.95 (0.89–1.02)
2.32–2.38	1378 (24.02)	32514 (23.53)	1.02 (0.95–1.10)
≥2.38	1164 (20.29)	27042 (19.57)	1.01 (0.93–1.09)
p-value for trend			0.30
**Age ≥50**			
**Calcium, mmol/L**			0.72 (0.53–0.96)
**Quartiles of calcium**			
<2.31	1034 (20.17)	14639 (18.16)	1.00 (Reference)
2.31–2.36	991 (19.33)	15987 (19.83)	0.87 (0.80–0.95)
2.36–2.44	1473 (28.74)	22749 (28.21)	0.93 (0.86–1.01)
≥2.44	1628 (31.76)	27257 (33.80)	0.89 (0.82–0.97)
p-value for trend			0.06
**Calcium according to age-specific cut-offs**			
Low	66 (1.29)	1004 (1.25)	1.20 (0.94–1.53)
Normal	4973 (97.02)	77765 (96.44)	1.00 (Reference)
High	87 (1.70)	1863 (2.31)	0.88 (0.71–1.09)
**Quartiles of albumin-corrected calcium ***			
<2.26	735 (14.34)	10481 (13.00)	1.00 (Reference)
2.26–2.32	1119 (21.83)	16969 (21.04)	0.93 (0.85–1.02)
2.32–2.38	1319 (25.73)	20579 (25.52)	0.92 (0.84–1.01)
≥2.38	1953 (38.10)	32603 (40.43)	0.92 (0.84–0.99)
p-value for trend			0.05

* Model not adjusted for albumin.

Table S3. Full-texts excluded from qualitative and quantitative analysis.

Study	Reason for Exclusion
Almquist et al. 2010 [1]	case-control study
Almquist et al. 2008 [2]	no data to investigate relationship between serum calcium and breast cancer
Belardi et al. 2013 [3]	serum calcium level measured post-diagnosis
Engel et al. 2010 [4]	serum calcium level measured post-diagnosis
Huss et al. 2014 [5]	investigates relationship between prediagnostic serum calcium levels and breast cancer mortality
Jacot et al. 2011 [6]	retrospective evaluation of patients with breast cancer
Martin et al. 2010 [7]	serum calcium level measured post-diagnosis
Pavithra et al. 2015 [8]	serum calcium level measured post-diagnosis
Proctor et al. 2010 [9]	no data to investigate relationship between serum calcium and breast cancer
Sahmoun et al. 2010 [7]	no data to investigate relationship between serum calcium and breast cancer
Schmidmayr et al. 2013 [10]	no data to investigate relationship between serum calcium and breast cancer
Uusi-Rasi et al. 2013 [11]	no data to investigate relationship between serum calcium and breast cancer
Wulaningsih et al. 2013 [12]	no data to investigate relationship between serum calcium and risk of breast cancer

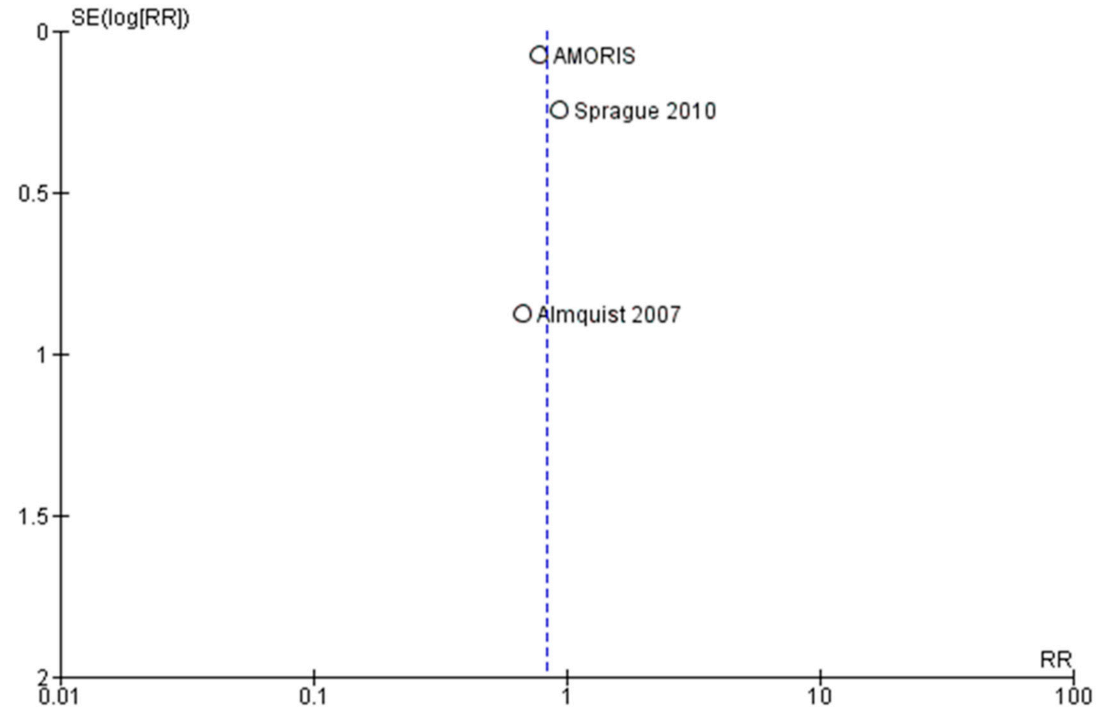

Figure S1. Funnel plot assessing risk of publication bias for studies reporting the association between serum calcium levels and breast cancer risk.

References

1. Almquist, M.; Bondeson, A.-G.; Bondeson, L.; Malm, J.; Manjer, J. Serum levels of vitamin D, PTH and calcium and breast cancer risk-a prospective nested case-control study. *Int. J. Cancer* **2010**, *127*, 2159–2168.
2. Almquist, M.; Bondeson, A.; Bondeson, L.; Halthur, C.; Malm, J.; Manjer, J. Reproductive history, lifestyle factors and season as determinants for serum calcium concentrations in women. *Scand. J. Clin. Lab. Investig.* **2008**, *68*, 777–785.
3. Belardi, V.; Fiore, E.; Giustarini, E.; Muller, I.; Sabatini, S.; Rosellini, V.; Seregni, E.; Agresti, R.; Marcocci, C.; Vitti, P.; et al. Is the risk of primary hyperparathyroidism increased in patients with untreated breast cancer ? *J. Endocrinol. Investig.* **2013**, *36*, 321–325.
4. Engel, P.; Fagherazzi, G.; Boutten, A.; Dupré, T.; Mesrine, S.; Boutron-Ruault, M.-C.; Clavel-Chapelon, F. Serum 25(OH) vitamin D and risk of breast cancer: a nested case-control study from the French E3N cohort. *Cancer Epidemiol. Biomark. Prev.* **2010**, *19*, 2341–50.
5. Huss, L.; Butt, S.; Borgquist, S.; Almquist, M.; Malm, J.; Manjer, J. Serum levels of vitamin D, parathyroid hormone and calcium in relation to survival following breast cancer. *Cancer Causes Control* **2014**, *25*, 1131–1140.
6. Jacot, W.P. Increased prevalence of vitamin D insufficiency in patients with breast cancer after neoadjuvant chemotherapy. *Breast Cancer Res. Treat.* **2012**, *134*, 709–717.
7. Martin, E.; Miller, M.; Krebsbach, L.; Beal, J.R.; Schwartz, G.G.; Sahmoun, A.E. Serum calcium levels are elevated among women with untreated postmenopausal breast cancer. *Cancer Causes Control* **2010**, *21*, 251–257.
8. Pavithra, V.; Sathisha, T.G.; Kasturi, K.; Mallika, D.S.; Amos, S.J.; Ragunatha, S. Serum levels of metal ions in female patients with breast cancer. *J. Clin. Diagn. Res.* **2015**, *9*, BC25–BC27.
9. Proctor, M.J.; Talwar, D.; Balmar, S.M.; O'Reilly, D.S.J.; Foulis, A.K.; Horgan, P.G.; Morrison, D.S.; McMillan, D.C. The relationship between the presence and site of cancer, an inflammation-based prognostic score and biochemical parameters. Initial results of the Glasgow Inflammation Outcome Study. *Br. J. Cancer* **2010**, *103*, 870–876.
10. Schmidmayr, M.E.; Lohmaier, J.; Richter, L.; Luppa, P.; Artmann, A.; Schuster, T.; Kiechle, M.; Seifert, V.R. The Endocrine Society' s 95th Annual Meeting and Expo, SAT-292 : Serum 25-Hydroxyvitamin D, Mammographic Density and Breast Biopsy Results. In Proceedings of the Endocrine Society's 95th Annual Meeting and Expo, San Francisco, CA, USA, 15–18 June 2013.
11. Uusi-Rasi, K.; Karkkainen, M.U.; Lamberg-Allardt, C.J. Calcium intake in health maintenance—A systematic review. *Food Nutr.* **2013**, *1*, 1–15.
12. Wulaningsih, W.; Holmberg, L.; Garmo, H.; Malmstrom, H.; Lambe, M.; Hammar, N.; Walldius, G.; Jungner, I.; van Hemelrijck, M. Prediagnostic serum inflammatory markers in relation to breast cancer risk, severity at diagnosis and survival in breast cancer patients. *Carcinogenesis* **2015**, *36*, 1121–1128.
